# Microfiltration results in the loss of analytes and affects the in vitro genotoxicity of a complex mixture of *Alternaria* toxins

**DOI:** 10.1007/s12550-020-00405-9

**Published:** 2020-08-14

**Authors:** Georg Aichinger, Natálie Živná, Elisabeth Varga, Francesco Crudo, Benedikt Warth, Doris Marko

**Affiliations:** 1 Department of Food Chemistry and Toxicology, Faculty of Chemistry, University of Vienna, Währinger Str. 38, 1090 Vienna, Austria; 2 Department of Food and Drug, University of Parma, Area Parco delle Scienze 27/A, 43124 Parma, Italy

**Keywords:** Emerging mycotoxins, Sample preparation, DNA damage, Risk assessment, Mass spectrometry, Public health

## Abstract

*Alternaria* molds produce a variety of chemically diverse secondary metabolites with potentially adverse effects on human health. However, data on occurrence in food and human exposure is inconsistent for some of these mycotoxins. Membrane filtration is a frequent step in many sample preparation procedures for LC-MS-based methods analyzing food contaminants. Yet, little is known about the possibility of adsorptive phenomena that might result in analyte losses. Thus, we treated a complex extract of *Alternaria* toxins with several types of syringe filters and unraveled the impact on its chemical composition by LC-MS/MS. We observed significant, and in some cases complete, losses of compounds due to filtration. Particularly, two key *Alternaria* toxins, alternariol (AOH) and its monomethyl ether (AME), were heavily affected. As a comparison with published food surveys indicating a correlation of the type of filtration used with lower incidence reports in food, our results point at a possible underestimation of AME in past exposure assessment. Also, perylene quinones were greatly affected by filtration, underlining the importance to take this into consideration during analytical method development. Furthermore, we applied the comet assay in HT-29 cells to elucidate the impact of filtration on the genotoxicity of the extract. We observed strong coincidences with the loss of epoxide-carrying metabolites and also an intriguing induction of oxidative DNA damage by yet toxicologically uncharacterized *Alternaria* toxins. In conclusion, we highlight potential issues with sample filtration and call for a critical re-evaluation of previous food occurrence data in the light of the results at hand.

## Introduction

Black molds of the genus *Alternaria* are ubiquitous microorganisms, which can grow on a variety of substrates, such as soil, decaying organic materials, and agricultural crops. The infestation of grains, vegetables, and fruits can lead to the occurrence of potentially toxic fungal secondary metabolites in food and feed (Ostry 2008).

**Fig. 1:**
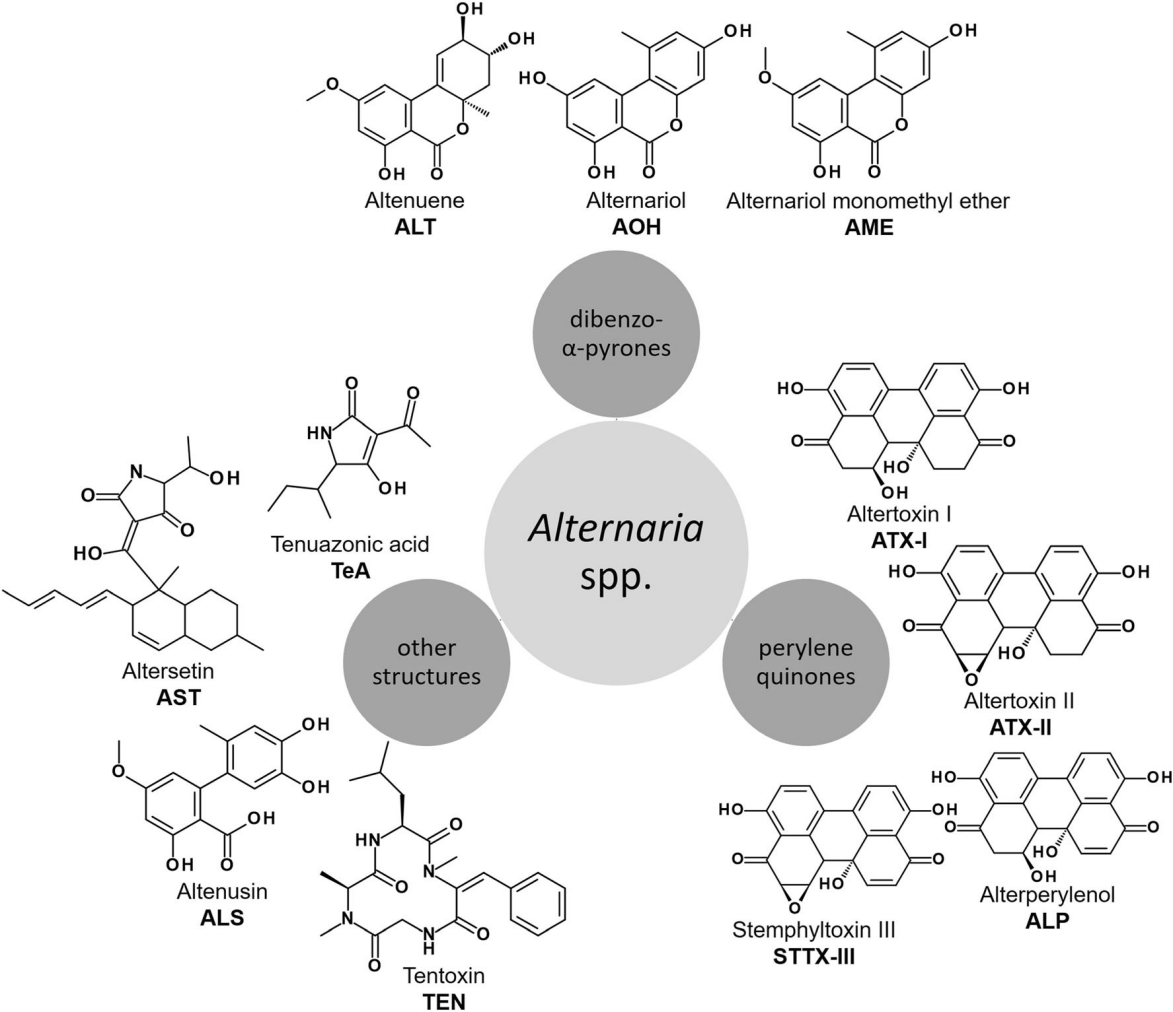
Chemical structures of selected *Alternaria* toxins

*Alternaria* molds are known to be able to produce more than 70 mycotoxins of a wide diversity of chemical classes (Fig. 1), which are until today not regulated by respective authorities and are thus considered to belong to the “emerging mycotoxins” (Fraeyman et al. 2017). Tenuazonic acid (TeA), an amine/amide metabolite, is the most frequently reported *Alternaria* toxin in food samples (Marin et al. 2013). Due to its rather mild acute toxic properties and lack of chronic toxicity, it is considered of little concern for humans (EFSA 2016).

However, two toxins belonging to the group of dibenzo-α-pyrones, namely alternariol (AOH) and alternariol monomethyl ether (AME), are considered a higher risk due to their potential impact on DNA integrity. Both toxins are very similar in chemical structure (Fig. 1) and biological activity. They are known to target human topoisomerase II, thus leading to DNA double-strand breaks in vitro (Fehr et al. 2009). Furthermore, they were reported to modulate inflammatory responses (Kollarova et al. 2018; Solhaug et al. 2015) and to interact with steroid receptors (Dellafiora et al. 2018; Frizzell et al. 2013; Lehmann et al. 2006; Stypuła-Trębas et al. 2017; Vejdovszky et al. 2017). Regarding their in vivo activity, the few available data are inconsistent and limited by technical problems which prevented clear evidence on systemic genotoxicity (Schuchardt et al. 2014). On this base, and the reported prevalence in food samples, the EFSA CONTAM panel lately estimated the 95th percentile dietary exposure of the European population to exceed toxicological threshold of concern (TTC) values (EFSA 2016).

Epoxide-carrying perylene quinones such as altertoxin (ATX)-II or stemphyltoxin-III (STTX-III) were demonstrated to possess a high potential for genotoxicity and other adverse effects in vitro (Aichinger et al. 2018; Del Favero et al. 2020; Fleck et al. 2012; Schwarz et al. 2012b; Tiessen et al. 2013). Even so, they have not yet been a primary focus of risk assessment, probably due to their high reactivity and thus low estimated bioavailability and relevance in vivo. However, perylene quinones were identified as the main contributors to DNA-damaging properties of extracts from *Alternaria* molds grown under laboratory conditions (Fleck et al. 2016; Schwarz et al. 2012b).

By applying single-cell gel electrophoresis, we recently reported ATX-II and STTX-III to be responsible for a good part of DNA damage caused by a naturally occurring mixture of *Alternaria* toxins in human cancer cells. However, a certain proportion of genotoxicity—particularly involving the induction of formamidopyrimidine-DNA glycosylase (FPG)–sensitive sites—was maintained after removing those two compounds from the mixture (Aichinger et al. 2019). Thus, other, yet toxicologically uncharacterized, *Alternaria* metabolites might cause DNA damage as well, which is a hypothesis that has urgently to be enlightened to ensure completeness of the respective risk assessment.

For assessing mycotoxins in biological matrices, state-of-the-art analytics mostly involve liquid chromatography coupled to tandem mass spectrometry (LC-MS/MS). This allows the simultaneous and sensitive quantification of multiple analytes (Warth et al. 2013). Many of these methods (Prelle et al. 2013; Siegel et al. 2010; Tölgyesi et al. 2015) use membrane filtration during sample preparation to reduce matrix effects, but primarily to protect LC systems from small particles.

However, it might be necessary to take possible adsorptive effects at membrane filters into account. Although related literature reports are rather limited, such a scenario does not seem unlikely, as previously published literature demonstrates the possibility of analyte-membrane interactions. For example, a study carried out by Carlson and Thompson (2000), who filtrated chemicals commonly encountered in drug preparations with cellulose, nylon, or polyvinylidene difluoride (PVDF) filters, found all analytes—and particularly those with rather acidic properties (e.g., sodium saccharine or salicylic acid)—to be adsorbed by the membrane matrices.

Mycotoxins, including AOH, were previously described to be absorbed by materials like cyclodextrins, which can be used to remove them from aqueous solutions (Fliszár-Nyúl et al. 2019). With materials commonly used for filtration steps during sample preparation, knowledge about such adsorptive effects is yet scarce for mycotoxins and other natural contaminants. However, the high chemical diversity of *Alternaria* toxins presupposes a considerable likelihood for such phenomena. To shed light on this issue would be of great importance for analytical method development and also for the evaluation of the analytical quality of previously published food surveys that used filtration techniques during sample preparation. Moreover, the high chemical reactivity of epoxide-carrying perylene quinones might cause an additional loss of those compounds due to the spontaneous formation of covalent adducts with filter membranes.

Thus, in this study, we used six different material types of syringe filters to assess the impact of filtration on the chemical composition of a complex mixture of *Alternaria* metabolites. Furthermore, we applied in vitro genotoxicity testing of the filtrates to collect hints on the identity of yet undescribed DNA-damaging mycotoxins.

## Materials and methods

### Materials

Ethidium bromide, Triton X-100, and solutions of NH_4_Ac (5 mM) and ammonia were purchased from Sigma-Aldrich (Schnelldorf, Germany). Normal and low-melting agarose were purchased from Bio-Rad (Frankfurt, Germany). LC-MS grade acetonitrile and methanol were purchased from Honeywell (Seelze, Germany). *Alternaria* toxins were purchased as reference materials: TeA, ATX-I, and tentoxin from Sigma-Aldrich (Steinheim, Germany), AOH and AME from Toronto Research Chemicals (Ontario, Canada), altenusin from Eubio (Vienna, Austria), altersetin from AnalytiCon Discovery GmbH (Potsdam, Germany). Altenuene was provided by Prof. Joachim Podlech (Institute of Organic Chemistry, Karlsruhe Institute of Technology, Germany). ATX-I, ATX-II, STTX-III, and alterperylenol were isolated from *Alternaria alternata* grown on rice by an optimized protocol based on Schwarz et al. (2012a). FPG was bought from New England Biolabs (Frankfurt, Germany). A complete extract (CE) originating from rice infected with the *Alternaria alternata* strain DSM 62010 was acquired as stated in Puntscher et al. (2019c) and Puntscher et al. (2019a).

Polytetrafluorethylene (PTFE, pore size 0.22 μm/diameter 30 mm), PVDF (0.45/15), and nylon (0.2/30) syringe filters were purchased from Carl Roth (Karlsruhe, Germany); polyethersulfone (PES, 0.2/30), glass fiber/cellulose acetate (GF/CA, 0.2/30), and regenerated cellulose (RC, 0.45/15) syringe filters were obtained from Sarstedt (Nümbrecht, Germany).

### Filtration

The ethanolic complete *Alternaria* toxin extract (CE) was diluted to 100 μg/mL in phosphate-buffered saline (PBS) solution (pH 7.4), resulting in a final concentration of approximately 0.04% ethanol. The resulting solution was used to perform filtration with six different syringe filters applying 3 mL each of the aqueous extract solution. The obtained aqueous filtrates were aliquoted and stored at − 80 °C for later genotoxic testing. For quantitative purposes, the filtrates were diluted by factors of 0, 10, or 70 with a 1:1 mixture of acetonitrile and methanol, vortexed, and subsequently injected into the LC-MS/MS system.

Since significant losses of compounds by filtration were observed (see Results section), the filtration experiment was repeated and after the filtration of the aqueous extract solution, the filters were washed with 3 mL of acetonitrile/methanol (1:1) to assess the recovery of potentially adsorbed toxins. The relative amount of toxins in the aqueous and the organic filtrate was determined by LC-MS/MS.

### LC-MS/MS

Aqueous and organic filtrates were measured by LC-MS/MS, applying a method originally developed for food matrices with minor modifications (Puntscher et al. 2018). In brief, a Dionex Ultimate 3000 ultra-high-performance liquid chromatography (UHPLC) system coupled to a TSQ Vantage triple quadrupole mass spectrometer (Thermo Scientific) was used. The MS was equipped with a heated electrospray ionization (HESI) interface (Thermo Scientific) and measurements were performed in the negative electrospray ionization mode. Chromatographic separation was performed using an Ascentis Express column (C18, 100 × 2.1 mm, 2.7 μm, Supelco, Sigma-Aldrich) equipped with a SecurityGuard™ (C18, 2 mm, Phenomenex, Aschaffenburg, Germany). The mobile phases consisted of 5 mM NH_4_Ac solution in water adjusted to a pH of 8.7 with 25% ammonia solution (eluent A) and methanol (eluent B). (For the detailed gradient, as well as mass transitions, please see Puntscher et al. (2018).) The mass transitions of AST can be found at Puntscher et al. (2019a).

### Cell culture

HT-29 colon carcinoma cells were acquired from the German Collection of Microorganisms and Cell Cultures (DSMZ, Braunschweig, Germany) and cultivated in Dulbecco’s Modified Eagle’s Medium (DMEM), supplemented with 10% (v/v) of heat-inactivated fetal calf serum and 1% (v/v) of penicillin/streptomycin. Dulbecco’s phosphate-buffered saline (DPBS), cell culture media, and supplements were obtained from GIBCO Invitrogen (Karlsruhe, Germany); cell culture flasks and dishes were purchased from Sarstedt (Nümbrecht, Germany). Cells were grown to 70–90% confluence at 37 °C in humidified conditions in 5% CO_2_ atmosphere.

### Single-cell gel electrophoresis (“comet assay”)

The comet assay was carried out following the guidelines of Tice et al. (2000) with minor modifications as published previously (Aichinger et al. 2017). Briefly, 150,000 cells were seeded in petri dishes and allowed to grow for 48 h. Cells were incubated for 1 h with the aqueous filtrates or the solvent control (PBS), diluted by a factor of 10 with non-supplemented DMEM. UV-B radiation was used to create a positive control. Cells were harvested, embedded in agarose, and lysed by immersion in a detergent-containing buffer. After treating half of the slides with FPG, electrophoresis (25 V, 300 mA) was performed under strong alkaline conditions (pH 13) for 20 min. Subsequently, slides were neutralized and stained with a solution of 10 μg/mL ethidium bromide. The “Comet Assay IV” system (Perceptive Instruments, Suffolk, UK) was used to score the tail intensity of 100 cells per object slide with a Zeiss Axioskop (*λ*_ex_ = 546 ± 1 nm; *λ*_em_ = 590 nm), the average of which was used as a measure for DNA damage.

### Statistical evaluation

Statistical analysis of results was performed using the Origin2019 software. Data was confirmed to be normally distributed with the Shapiro-Wilk test. Subsequently, we carried out calculations for assessing significant differences using either one-way ANOVA with Fisher LSD post hoc testing, or Student’s *t* test.

## Results

### Chemical composition

Filtration of the CE with different syringe filters resulted in a diverse pattern of compound loss depending on both the type of filter and the analyte (Table 1). The potentially hazardous dibenzo-α-pyrones AOH and AME were significantly reduced by RC and completely removed by nylon, PES, or GF/CA filtration. A prominent difference between the two compounds appeared with PVDF membranes, as it adsorbed AME to a much higher extent (almost completely) as compared with AOH (24% reduction). The structurally similar altenuene (ALT) was seemingly less affected by filtration, with recoveries of 85% for PVDF, 29% for nylon, 18% for PES, 59% for GF/CA, and 103% for RC membranes. Tentoxin (TEN) was moderately affected by all filter types, with losses between 40% (PES) and 60% (GF/CA). Of particular interest, the perylene quinone family—ATX-I and ATX-II, alterperylenol (ALP), STTX-III—exerted similar adsorptive properties. They were not susceptible to PTFE or RC treatment and moderately impacted by PVDF, but almost completely eliminated by nylon, PES, and GF/CA filtration. The less studied metabolites altersetin (AST) and altenusin (ALS) showed distinct adsorptive behaviors. AST was moderately affected by PTFE, significantly reduced by RC, and completely eliminated by PVDF, nylon, and PES membranes. ALS was almost not affected by PTFE, PVDF, GF/CA, and RC filtration, but significantly adsorbed at nylon and PES membranes.

**Table 1:** Influence of microfiltration on the chemical composition of a complex mixture of *Alternaria *toxins, as obtained by LC-MS/MS analysis. Concentrations are provided in micrograms per liter or milligrams per liter except for STTX-III, where absolute quantification was not possible due to a lack of a reference standard; thus, the respective peak area units (AU) are presented

	AOH (μg/L)	AME (μg/L)	ALT (μg/L)	TeA (mg/L)	TEN (μg/L)	ATX-I (mg/L)	ATX-II (mg/L)	ALP (mg/L)	STTX-III (10^6^ AU)	ALS (μg/L)	AST (mg/L)
Unfiltered	90.3	88.3	89.2	62.5	1.5	1.04	1.38	1.38	680	25.6	2.23
PTFE	84.4	72.7	83.3	60.1	0.9	1.05	1.35	1.30	700	23.3	1.37
PVDF	68.9	< 0.06	75.8	58.0	0.8	0.79	0.69	1.03	254	25.8	< 0.0002
Nylon	< 0.3	< 0.06	26.0	56.3	0.8	< 0.00024	< 0.001	< 0.001	5	8.9	< 0.0002
PES	< 0.3	< 0.06	15.7	59.3	0.9	< 0.00024	< 0.001	< 0.001	3	7.4	< 0.0002
GF/CA	< 0.3	< 0.06	52.7	57.9	0.6	0.0035	< 0.001	0.006	9	23.6	1.10
RC	42.9	38.8	91.7	59.1	0.7	0.98	1.34	1.32	688	23.3	0.71

### Recovery of adsorbed toxins

The used filters were washed with methanol/acetonitrile (1:1) to allow for an estimation of whether organic solvents could recover the adsorbed toxins. A total recovery was calculated by summing up the peak areas measured in aqueous and organic filtrates, taking into account respective dilution factors and filtrate volumes. Of note, a certain error should be expected to occur due to unavoidable residues of the aqueous filtrate in the syringe filters after the first filtration step, as well as slight variations in passage volumes. Thus, it is important to state that the obtained recovery values should be considered as approximation.

**Fig. 2:**
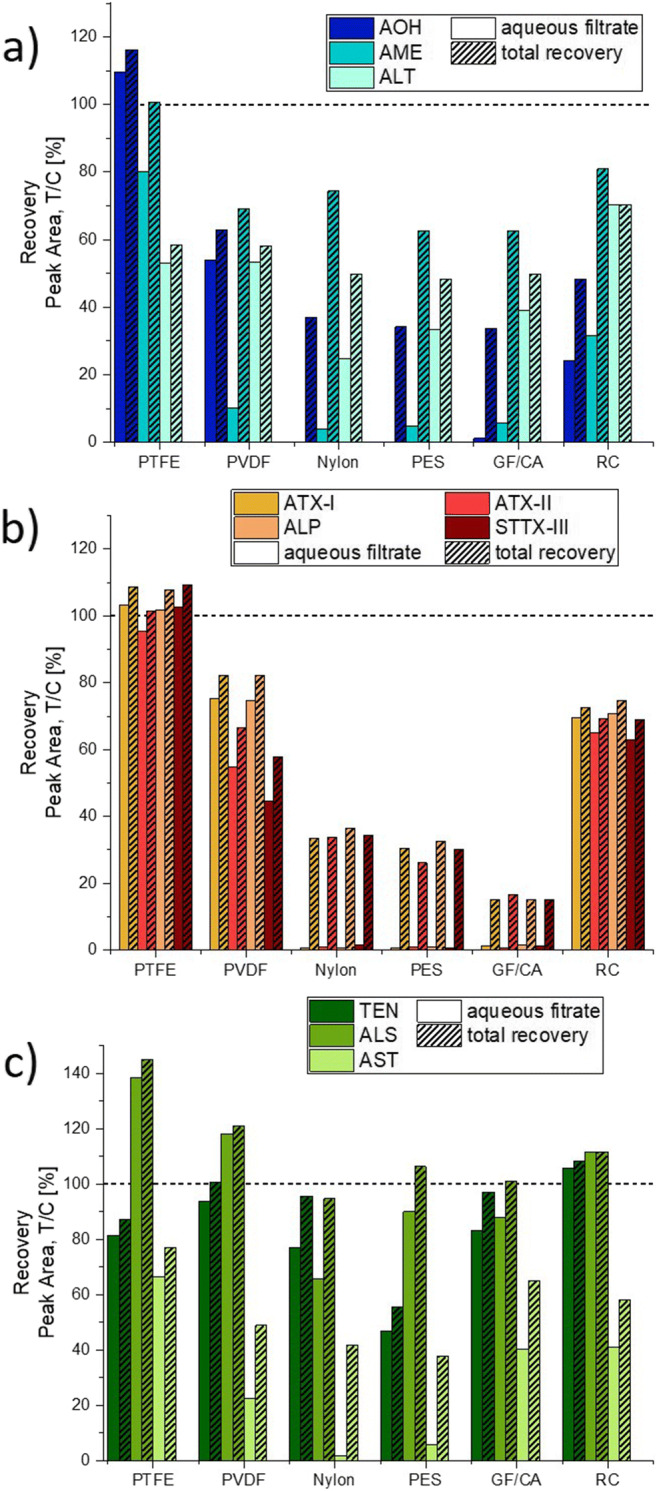
Recovery of (a) dibenzo-α-pyrones, (b) perylene quinones, (c) the miscellaneous toxins TEN, AST, and ALS after aqueous filtration and an additional washing step with methanol/acetonitrile (1:1). Bars show peak areas, corrected for dilution factors and sample volumes, and related to the unfiltered sample, which is indicated by a dashed line

The respective results revealed the ability of methanol/acetonitrile to elute part of the adsorbed mycotoxins (Fig. 2), at least for some toxins. Roughly a third of the lost AOH was recovered from nylon, PES, GC/CA, and RC filters. The total recovery for AME from the PVDF membrane was about 50% higher as compared with the aqueous filtrate, but AOH was not washed from this filter at all (Fig. 2(a)). Furthermore, approximately 50% of the adsorbed AME were recovered from nylon, PES, GC/CA, and RC filters (Fig. 2(a)). The total recovery of ALT was comparable with the other dibenzo-α-pyrones, with the exception that the recovered level from PTFE filters did not exceed 58% even after organic elution (Fig. 2(a)). The perylene quinones ATX-I, ATX-II, ALP, and STTX-III showed very similar recovery patterns (Fig. 2(b)). All of them were partially (25–36%) eluted from nylon and PES and to a lesser extent (14–16%) from GF/CA filters, while the washing of PVDF and RC filters had little to no effect on the total recovery (< 8%). Small amounts of TEN were elutable from nylon and GF/CA, but not from PTFE filters (Fig. 2(c)). Regarding ALS, 29% of the adsorbed toxin was recovered by washing the nylon filter, 13% from GF/CA, 16% from PES, and 7% from PVDF filters. Of note, the compound adsorbed at the PTFE membrane was again only recovered in insignificant doses (Fig. 2(c)). Furthermore, moderate amounts of AST (11–40%) were recovered from all filters (Fig. 2(c)).

### Genotoxicity

**Fig. 3:**
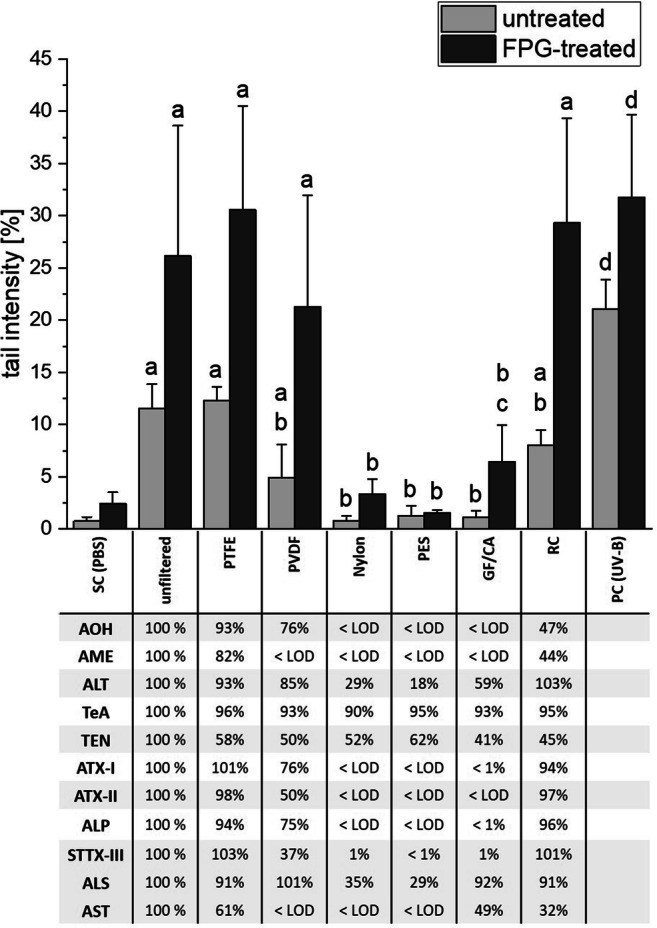
Impact of filtration on the genotoxicity of the *Alternaria* extract in HT-29 cells, as measured by comet assay. Bars are depicting mean values + SD of the measured tail intensities of at least 4 independent biological replicates. Normal distribution was confirmed with the Shapiro-Wilk test. Significant differences were calculated by one-way ANOVA, followed by Fisher LSD post hoc testing (*p* < 0.05), with “a” indicating a significant difference to the respective solvent control (SC) and “b” to the unfiltered extract. A separate one-way ANOVA was carried out for the samples revealing very low tail intensities to again test for significant differences to the respective solvent control, which are indicated by “c.” The positive control was tested against the SC with Student’s *t* test, observed significant differences therewith are indicated by “d.” The same was used for testing the impact of FPG treatment for each sample, which significantly enhanced tail intensities of all samples, including SC, except for the PES filtrate (not indicated). The underlying table shows LC-MS data for the corresponding aqueous filtrates, in relation to the respective toxin concentration in the unfiltered sample

DNA damage was assessed by conducting comet assays after a 1 h incubation of HT-29 cells with 1:10 dilutions of the aqueous filtrates or the unfiltered extract in DMEM, resulting in a final concentration of 5 μg/mL. Each sample was measured with and without FPG treatment to discriminate for the induction of FPG-sensitive sites. Tail intensities (TI) for both the solvent (SC) and the positive control were in line with our expectations and confirmed the functionality of our test system (Fig. 3). In line with recently published data (Aichinger et al. 2019), the unfiltered extract caused mean TIs of 11.5% (untreated) and 26.1% (+ FPG). Filtration of the CE with PTFE did not have an impact on its genotoxicity. Filtrations with PVDF or RC reduced the induction of DNA strand breaks without enzymatic treatment to 4.9% and 8.0% TI, respectively, but did not impact the overall genotoxicity including FPG-sensitive sites. The strongest detoxifying effects were observed for filtrations with nylon, PES, and GF/CA membranes. In these cases, the levels of strand breaks were reduced to not being distinguishable from the SC independent of FPG treatment. However, re-running one-way ANOVA only for the low-damaged groups (SC, nylon, PES, GF/CA) revealed the GF/CA-treated filtrate to induce significantly higher genotoxicity as compared with the SC, after enzymatic treatment.

## Discussion

Syringe filters are widely applied in sample preparation for LC-MS based food safety surveys, yet little is known about possible interactions between the membrane material and potential analyte losses. Crop-infesting *Alternaria* spp. may produce a cocktail of more than 70 toxic metabolites for which up to date no regulations exist and which are thus commonly classified as “emerging mycotoxins” (Gruber-Dorninger et al. 2017). The high chemical diversity in this mixture of contaminants gives rise to the suspicion that at least some of its substances might be affected by adsorptive phenomena during filtration. If occurring during food monitoring studies, this could lead to an underestimation of humane exposure with potentially problematic consequences for risk assessment. Also, knowing such interactions would be of great value for the future development of appropriate analytical methods for *Alternaria* toxins.

To address these pending questions, we used a well-characterized complex extract from an *Alternaria alternata* strain grown on rice (Puntscher et al. 2019c) and subjected it to microfiltration by using six distinct syringe filter types. The subsequent analysis of the filtrates’ chemical composition by LC-MS/MS revealed significant adsorptive phenomena that vary with both the membrane material and the monitored analyte (Table 1).

TeA, the most abundant *Alternaria* toxin, was not adsorbed in significant proportions at any of the tested filters. The genotoxic compound AOH, which is of particular concern for human health according to EFSA (2016), was not affected by PTFE filtration, but partially adsorbed by PVDF and RC and completely bound by nylon, PES, and GF/CA filters. Of note, we found filtration to affect the structurally and mechanistically similar derivative AME in a stronger way, as the toxin was removed completely by filtration with PVDF and even adsorbed to a minor extent by PTFE membrane (Table 1). The lost dibenzo-α-pyrones could only partially be recovered by washing the filters with acetonitrile/methanol (Fig. 2), indicating the relevance of adsorptive phenomena also in these eluents.

Interestingly, and potentially of concern, there seems to be a connection between our results and published occurrence data sets, in which food surveys employing filtration with PVDF or nylon filters report conspicuously less frequent contaminations with AOH and AME as compared with studies using PTFE, RC, or no filters at all (Table 2). Moreover, studies screening food types particularly affected by *Alternaria* toxin contamination, e.g., tomato products, tend to report AOH, but no AME at all, when using PVDF filters (De Berardis et al. 2018; Prelle et al. 2013). However, in the absence of filtration, the latter compound is commonly reported to occur even more frequently and in higher concentrations as compared with AOH (Noser et al. 2011; Puntscher et al. 2019b; Zhao et al. 2015).

**Table 2:** Overview of recently (< 10 years) reported *Alternaria* toxin occurrence data in food as determined by LC-MS multi-analyte methods, grouped by conducted type of microfiltration. “n.d.” (not detected) indicates the respective toxin was included in the survey, but not found in any of the analyzed samples at levels above the LOD

Filtration	Published by	Food matrices	Specification	*Alternaria* toxins include and % of positive samples						
				AOH	AME	TeA	TEN	ALT	ATX-I	ALP
PTFE	Janić Hajnal et al. (2015)	Wheat	-	12	6	69	-	-	-	-
	López et al. (2016)	Wine, cereals, apple/tomato-based products, dried fruit, sunflower seeds, and seed oil	Overall	7	5	22	15	n.d.	-	-
			Tomato sauces	50	50	100	n.d.	n.d.	-	-
	Puntscher et al. (2019b)	Wheat flour		13	40	31	18	-	20	36
PVDF	De Berardis et al. (2018)	Tomatoes and fruit-based products	-	n.d.	n.d.	18	n.d.	-	-	-
	Prelle et al. (2013)	Apple juices, beers, tomato sauces, olives, dried basil	Overall	24	n.d.	3	3	9	-	-
			Tomato sauces	50	n.d.	n.d.	10	80	-	-
Nylon	Rodriguez-Carrasco et al. (2016)	Tomatoes/tomato-based products	-	13	7 (< LOQ)	-	n.d.	-	-	-
	Rubert et al. (2012)	Barley	-	n.d.	-	-	-	n.d.	-	-
	Juan et al. (2016)	Strawberries	-	25	19	-	n.d.	-	-	-
RC	Hickert et al. (2016)	Tomato products, bakery products, sunflower seeds, fruit juices, vegetable oils	Tomato products	71	79	91	26	n.d.	n.d.	-
Unspecified	Gotthardt et al. (2019)	Infant foods	-	32	92	89	84	n.d.	16	8
	Diana Di Mavungu et al. (2009)	Food supplements	-	n.d.	n.d.	-	-	n.d.	-	-
	Ssepuuya et al. (2018)	Sorghum	-	2.5	1.6	-	-	0.07	-	-
	Walravens et al. (2016)	Tomato products, fruit/vegetable juices	Tomato products	71	54	100	64	50	n.d.	-
None	Noser et al. (2011)	Tomatoes/tomato-based products	-	27	26	81	10	2	n.d.	-
	Qiao et al. (2018)	Cherries/cherry-based products	-	35	42	76	49	31	-	-
	Zhao et al. (2015)	Tomato- and citrus-based products	Citrus-based food or fresh tomatoes	n.d.	n.d.	n.d.	n.d.	-	-	-
			Tomato products	35	88	100	73	-	-	-
	Puntscher et al. (2019b)	Tomato sauces, sunflower seed oil	Tomato products	30	55	71	11	-	23	21

This discrepancy might indicate a potential filtration-derived underestimation of AME’s risk for human health. In their last dietary exposure assessment, members of the EFSA CONTAM panel stated that “reported levels of AME were lower than those reported for AOH” and that AME was “quantified in a few samples of tomato-based products, although at the lower levels than those reported for AOH” (EFSA 2016). The results of the study at hand suggest that the respective occurrence data should be critically re-evaluated on the basis of used filtration techniques and this step should be particularly well-monitored during future method development and validation. In the light of this, accurate specification of the used membrane filters is crucial for data interpretation and should be further encouraged (Table 2). Providing manufacturer and a respective trademark is not sufficient since this does not define the filter material and, e.g., PTFE and PCDF filters exert significant differences in their adsorption of analytes.

Another particularly intriguing question arising from our results is whether the described adsorptive phenomena could be responsible for the rare reports of mycotoxins belonging to the perylene quinone family in food samples. Despite their low occurrence data in food, they are evidently among the predominantly formed secondary metabolites of *Alternaria* molds grown under laboratory conditions (Puntscher et al. 2019c; Zwickel et al. 2018). For epoxide-carrying representatives like ATX-II and STTX-III, the chemical reactivity towards co-occurring food constituents and fibers might at least contribute to this mystery (Aichinger et al. 2018; Crudo et al. 2020). However, for non-reactive metabolites like ATX-I or ALP, our data suggests that filtration might be a major source of compound loss. Even the considerable concentrations present in the used extract were completely lost by filtration with nylon, PES, and GF/CA filters, and significantly reduced by PVDF filtration (Table 1). Furthermore, the absence of differences in the adsorptive behavior between epoxide and non-epoxide containing perylene quinones, and the possibility to recover parts of all the compounds by washing with organic solvents (Fig. 2), rules out a chemical reaction of ATX-II/STTX-III with membrane materials.

Our data underlines the importance of considering filtration with caution when screening for these compounds. In line with this concern, so far, the only report of ATX-II in naturally contaminated food samples applied a sample preparation protocol without a filtration step (Puntscher et al. 2020). Nevertheless, and on the contrary to AOH/AME, a clear connection between filtration technique and reporting in food samples could not be established from the available data, probably also due to the little number of studies incorporating methods which include those toxins.

An additional aim of this study was to assess the impact of filtration on the DNA-damaging properties of the used complex extract, as previous experiments suggested a contribution of compounds yet not characterized as genotoxic agents (Aichinger et al. 2019). Using HT-29 colon carcinoma cells, we performed single-cell gel electrophoresis to observe a pattern of genotoxicity that was largely following our expectations based on the measured chemical composition of the filtrates (Fig. 3). In line with previous data, DNA damage—both with and without FPG treatment—seemed proportional to the present concentration of ATX-II and STTX-III, the two most potent genotoxic chemicals of the extract (Fleck et al. 2016; Schwarz et al. 2012b). No significant induction of DNA strand breaks was determined for any of the filtrates hardly containing those compounds (nylon, GF/CA, PES). Thus, additional genotoxic compounds can be expected to exert similar adsorptive properties and could possibly be structurally related, such as ATX-III that was not included in the analytical method, and thus may be present in the extract in unknown concentrations.

Intriguingly, the GF/CA-treated extract was still able to significantly induce FPG-sensitive sites (Fig. 3), indicating the presence of compounds responsible for oxidative stress. With AOH, AME, and the perylene quinones practically absent and TeA and TEN concentrations not being higher than in PES-treated filtrates, the possible identifiable candidates to exert this effect are limited to ALT, ALS, and AST. ALT was previously analyzed in the comet assay with respect to DNA-damaging potential in HT-29 cells and showed no effect up to a concentration of 100 μM (Fehr et al. 2009). Data on the impact of AST and ALS on human cells are scarce, with AST—the compound occurring in much higher concentrations in our extract (Table 1)—being described as a weak topoisomerase II inhibitor under cell-free conditions (Jarolim et al. 2016). Its potential to induce oxidative stress and possibly oxidative DNA damage should be examined by further studies. However, it also cannot be excluded that the observed induction of FPG-sensitive sites was caused by further, yet completely unknown, *Alternaria* metabolites.

Taken together, this study demonstrates the high potential of adsorptive filter-analyte interactions, which might result in an underestimation of toxin occurrence, and thus exposure, of certain *Alternaria* toxins, particularly the genotoxic AME as well as perylene quinones. We conclude that both the future LC-MS/MS method development and the retrospective data evaluation in the course of risk assessment should take such phenomena into consideration. Furthermore, we collected additional hints on yet undescribed genotoxic *Alternaria* metabolites and recommend respective testing, including AST.

## Data Availability

All data and existing sample material will be stored at the Department of Food Chemistry and Toxicology, University of Vienna, and can be made available upon reasonable request.

## Abbreviations

ALP: Alterperylenol
ALS: Altenusin
ALT: Altenuene
AME: Alternariol monomethyl ether
AOH: Alternariol
AST: Altersetin
ATX: Altertoxin
FPG: Formamidopyrimidine-DNA glycosylase
GF/CA: Glass fiber/cellulose acetate
PES: Polyethersulfone
PTFE: Polytetrafluorethylene
PVDF: Polyvinylidene difluoride
RC: Regenerated cellulose
STTX: Stemphyltoxin
TeA: Tenuazonic acid
TEN: Tentoxin
TI: Tail intensity
